# The Roles of Non-retinotopic Motions in Visual Search

**DOI:** 10.3389/fpsyg.2016.00840

**Published:** 2016-06-01

**Authors:** Ryohei Nakayama, Isamu Motoyoshi, Takao Sato

**Affiliations:** ^1^Department of Psychology, The University of TokyoTokyo, Japan; ^2^Department of Life Sciences, The University of TokyoTokyo, Japan

**Keywords:** visual search asymmetry, search efficiency, motion coordinate, spatiotopic motion, relative motion, smooth pursuit eye movement

## Abstract

In visual search, a moving target among stationary distracters is detected more rapidly and more efficiently than a static target among moving distracters. Here we examined how this search asymmetry depends on motion signals from three distinct coordinate systems—retinal, relative, and spatiotopic (head/body-centered). Our search display consisted of a target element, distracters elements, and a fixation point tracked by observers. Each element was composed of a spatial carrier grating windowed by a Gaussian envelope, and the motions of carriers, windows, and fixation were manipulated independently and used in various combinations to decouple the respective effects of motion coordinate systems on visual search asymmetry. We found that retinal motion hardly contributes to reaction times and search slopes but that relative and spatiotopic motions contribute to them substantially. Results highlight the important roles of non-retinotopic motions for guiding observer attention in visual search.

## Introduction

The efficiency of visual search depends on the relative strength of feature properties between target and distracters. For instance, motion is a dominant feature in visual search as a moving target among stationary distracters is detected more rapidly and more efficiently (i.e., a flatter function of reaction time vs. display set-size) than a stationary target among moving distractors (Royden et al., 2001). This asymmetry is attributed to a strong perceptual saliency of visual motion signals (Theeuwes, 1994, 1995; Rosenholtz, 1999; Wolfe, 2001) and an ability of motion signals to immediately capture observer attention (Hillstrom and Yantis, 1994; Abrams and Christ, 2006).

In many studies on visual search, motion is defined in a retinal coordinate system as a positional change over time on the retinal surface. However, other motion coordinate systems are possible and relevant to visual inference and human vision including relative motion (the motion of an object relative to other objects or a background scene) and spatiotopic motion (the motion of an object relative to the observer's body or head). Motion signals from these three coordinate systems—retinal, relative, and spatiotopic—are involved in everyday motion perception, but their respective perceptual contribution can only be teased apart through careful experimental manipulation. For example, if an observer is asked to fixate on a static point in space as an object moves through that space, then the object moves in all three coordinate systems. However, if an observer is asked to fixate on a moving object and tracks it using smooth pursuit, then the object remains stationary on the observer's retina but moves with respect both to background objects and to the observer's body. The neural representation of retinal motion signals originates primarily from retinal input, but the representation of non-retinal motions signals are generated by the comparison and integration of retinal inputs or with sensorimotor signals (Wurtz, 2008).

Abundant evidence shows that the visual system possesses neural mechanisms tuned to motions in non-retinal coordinates. Psychophysical and electrophysiological studies support the existence of neural sensors detecting relative motions (Allman et al., 1985; Born and Tootell, 1992), and observations from some of the literature's classics studies referred to the effects of eye and body movement on motion perception (e.g., Fleischl, 1882; Aubert-Fleischl phenomenon, Aubert, 1887; Filehne illusion, Filehne, 1922). More recently, visual processing in spatiotopic coordinates was investigated by analyzing the motion or pattern perception during smooth pursuit eye movements (Schütz et al., 2007, 2008; Terao and Murakami, 2011). Electrophysiological studies have also found cells that specifically respond to spatiotopic motions in cortical areas such as V3A (Galletti et al., 1990), MST (Erickson and Thier, 1991; Chukoskie and Movshon, 2009), and 7a (Sakata et al., 1985).

Based on the findings mentioned above, it is likely that visual search is enhanced by relative and spatiotopic motions that involve higher-order motion processing as well as retinal motion. To test for this possibility, the present study revisited the search asymmetry of the moving/stationary targets paradigm with the intent of measuring the separate contributions of the retinal, relative, and spatiotopic motion signals to visual search performance.

**Figure 1:**
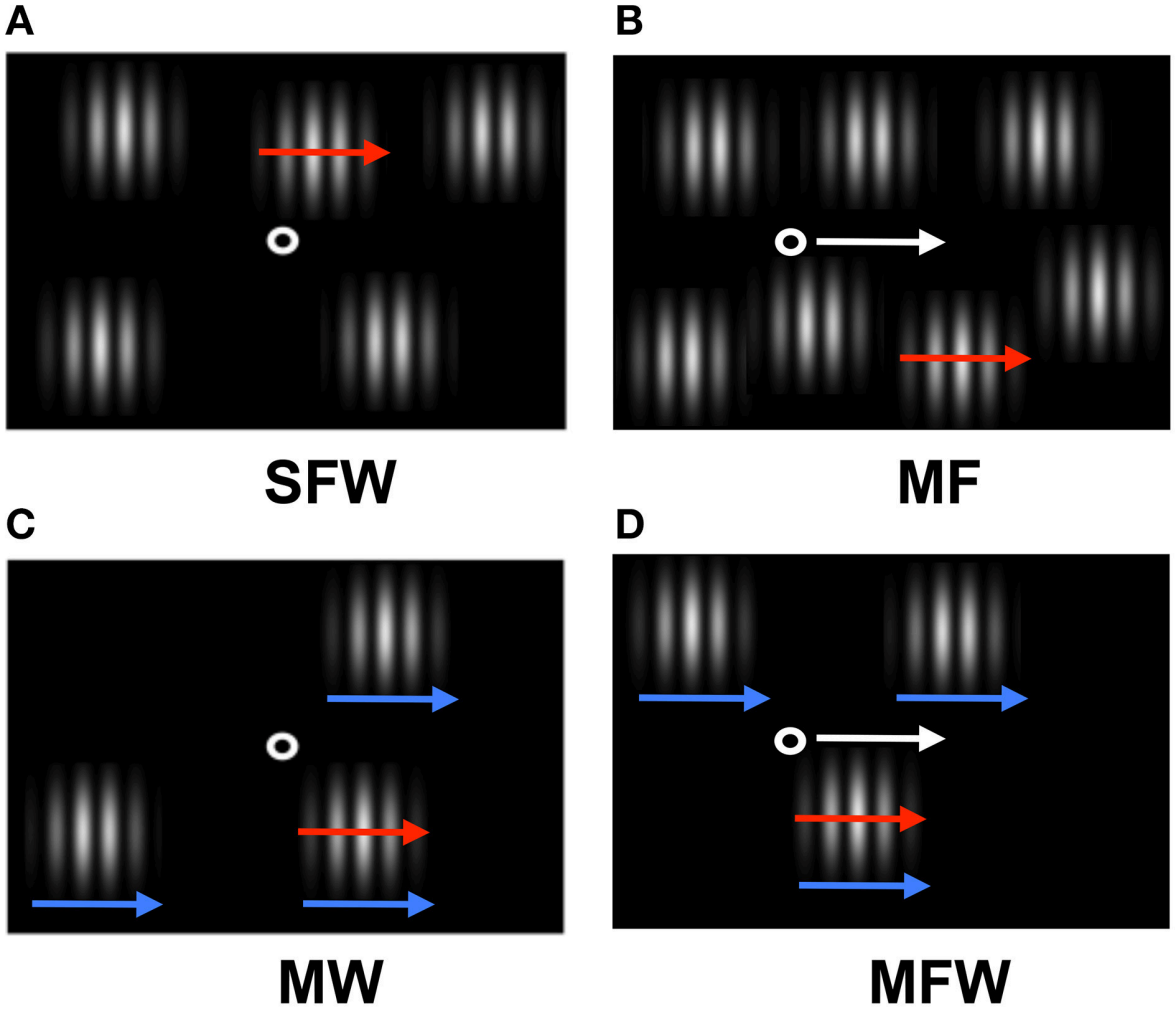
**Schematic of the stimulus display used in the experiment**. Red arrows indicate target grating motion (which can be assigned to distracter gratings depending on experimental condition), white arrows indicate motion of the fixation point, and blue arrows indicate spatial windows motion (see Supplementary Materials). **(A)** Target grating drifts but fixation point and windows remain stationary (stationary fixation/window, SFW condition). This condition produces motion in all three coordinate systems (retinal, relative, and spatiotopic). **(B)** Target grating drifts while the observer tracks a fixation point moving together with the target grating (moving fixation, MF condition). In contrast to **(A)**, the target grating remains stationary on the retina and motion is present only in relative and spatiotopic coordinates. **(C)** The windows move together with the drifting target grating while the fixation point remains stationary (moving window, MW condition). In contrast to **(A)**, the relative motion between the target grating and windows is eliminated and motion is present only in retinal and spatiotopic coordinates. **(D)** Both the fixation point and the windows move together with the drifting grating while the observer tracks fixation (moving fixation/window, MFW condition). Here, motion exists only in the spatiotopic domain.

We devised a simple stimulus (Figure 1) that enabled us to examine the respective effects of three types of motion—retinal, relative, and spatiotopic—on visual search. The stimulus consisted of a fixation point (moving or stationary) and several elements, each of which was composed of a spatial grating pattern (moving or stationary) windowed by a Gaussian envelope window (moving or stationary). In target-present trials, one of the grating patterns (the target) moved horizontally while other gratings (the distracters) were kept stationary, or alternatively one of gratings (the target) remained stationary while the others (the distracters) moved horizontally. In target-absent trials, all grating patterns were either stationary or moving in the same direction. While the fixation point, gratings, and windows can move independently of each other in principle, we added a constraint such that fixation and envelopes either all had to remain stationary or all had to move together horizontally depending on specifics on the chosen condition.

In the first condition (stationary fixation/window, or SFW), the drifting grating produced motion in all coordinate systems (retinal, relative, and spatiotopic) as fixation and windows remained stationary (Figure 1A). In the second condition (moving fixation, or MF), observers tracked a fixation point that moved together with the drifting grating while windows remained stationary (Figure 1B). In this condition, the drifting grating remained stationary in retinal coordinate only but drifted both in relative and in spatiotopic coordinates. In the third condition (moving window, or MW), windows moved together with the drifting grating while fixation remained stationary (Figure 1C). In this case, the drifting grating remained stationary in relative coordinates but moved in relationship to the retina and with respect to the observer. In the fourth condition (moving fixation/window, or MFW), both fixation and windows moved together with respect to the drifting grating (Figure 1D). Here, the drifting grating remained stationary with respect to the retina and windows but drifted in relationship to the observer. By using these four different stimulus configurations, we were able to dissociate the motion physics of the three coordinate systems and to quantitatively estimate the relative contributions of motion signals from these coordinate systems to the search asymmetry of moving/stationary targets.

## Methods

### Observers

Six naive participants and one of the authors (RN), with corrected-to-normal vision, participated in the experiment. All experiments followed the Declaration of Helsinki guidelines, and all observers provided informed consent.

### Apparatus

Images were generated by a personal computer using the Psychophysics toolbox (Brainard, 1997; Pelli, 1997) and MATLAB (Mathworks, Inc.), and displayed on a gamma-corrected 21-inch CRT (Mitsubishi Diamondtron M2 RDF223G; 800 × 600 pixel) through a video attenuator (Bits++, Cambridge Research Systems Ltd.) with a frame rate of 150 Hz. The pixel resolution of the CRT was 3.0 min/pixel at the viewing distance of 57 cm. The front screen of the CRT together with the frame of display was covered by a neutral-density (ND) film of 23% transmittance so that the background luminance in the screen was 0.012 cd/m^2^ and the frame of display was kept almost completely invisible. Throughout the experiment, movements of both eyes were monitored by means of Viewpoint Eye Tracker (220 Hz; Arrington Research, Inc.).

### Stimuli

The search display was composed of vertical grating patterns (spatial frequency of 1.0 cycle/deg) defined by a sinusoidal luminance modulation around the mean of 10.5 cd/m^2^. The luminance of the grating was spatially windowed by a Gaussian window (SD: 0.8°) such that grating edges would smoothly fade out into the low-luminance background (Figure 1). A fixation point (0.6° in diameter, 20.1 cd/m^2^) was presented either at the center of the screen if stationary or, if moving, at a location horizontally-shifted from the center by half the distance of the fixation point's total displacement. Grating patterns were randomly laid out spatially such as not to overlap with each other with the constraint that patterns remained within 10° of fixation at the halfway point of each trial if the fixation or window moved. Set-size varied from three to nine grating stimuli between trials.

Target and distracter gratings were either stationary or moving and distracters differed from each other only in their respective motions: the target grating drifted at 6°/s while distracter gratings remained stationary, or the target grating remained stationary while distracter gratings drifted at 6°/s. Henceforth, a moving grating will be called a “MOVING grating” since, although it physically moved on the CRT screen, it may have remained stationary with respect to the relative and/or spatiotopic coordinates systems owing to experimental manipulation. Similarly, a grating that remained stationary on the CRT will be called a “STATIONARY grating.” The direction of grating motion (either leftward or rightward) and whether either the target or the distracters were assigned to the MOVING condition, were both randomly counterbalanced across trials. The motion of fixation point and spatial windows were yoked such that either fixation and windows all remained stationary or fixation and windows all moved in tandem depending on the experimental condition. When fixation and windows moved, they did so with the same velocity and in the same direction as the MOVING grating.

There were four combinations of the stationary/moving parameters for the fixation point and spatial window. The first combination was the SFW condition (Figure 1A) where both fixation and the spatial window were stationary and the MOVING grating produced motion in all three coordinate systems: retinal, relative, and spatiotopic. This condition was created to test the classical motion effect on search asymmetry of moving/stationary targets. The second combination was the moving fixation (MF) condition (Figure 1B) where fixation moved in the same direction and with the same velocity as the MOVING grating. In this condition, the MOVING grating remained stationary in retinal coordinates (due to the observer's eyes engaging in smooth-pursuit tracking of the fixation point) produced motion signals in relative and spatiotopic coordinates. The third combination was the moving window (MW) condition (Figure 1C) where the spatial window moved in the same direction and with the same velocity as the MOVING grating while fixation remained stationary. In this condition, the MOVING grating moved in retinal and spatiotopic coordinates but remained stationary in relative coordinates. The last combination was the MFW condition (Figure 1D) where both fixation and the spatial window moved and the observer tracked the moving fixation point. In this condition, the MOVING grating produced motion only in the spatiotopic domain because retinal and relative motions were canceled by eye-movement tracking and the motion of the spatial window. The type of motion (retinal, relative, and spatiotopic) involved with both MOVING and STATIONARY gratings are graphically represented in Table 1 for each experimental condition.

**Table 1:** **Three types of motion observed for each experimental condition**.

	**Moving grating**			**Stationary grating**		
**Spatial coordinates of motion**	**R**	**Rel**	**S**	**R**	**Rel**	**S**
Stationary fixation/window	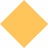	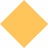	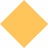	—	—	—
Moving fixation	—	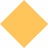	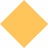	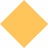	—	—
Moving window	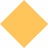	—	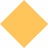	—	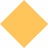	—
Moving fixation/window	—	—	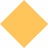	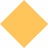	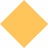	—

“R” represents retinal coordinates, “Rel” represents relative coordinates, and “S” represents spatiotopic coordinates. Yellow diamonds denote the presence of the corresponding type of motion involved, respectively, in the MOVING grating and the STATIONARY grating, which was randomly assigned to target/distracter.

### Procedure

The experiment was conducted in a dark room, and each condition was tested in separate random-ordered blocks. For each condition, the target was present in half of each block's trials and absent in the other half. In each trial, all stimuli remained stationary for 1 s after which time the designated components moved for 3 s. The observer was required to keep a steady gaze on fixation or to track the fixation point as accurately as possible when it moved. To facilitate the tracking of a moving fixation point, fixation disappeared prior to its initial motion and reappeared at a position shifted by 1.2° toward the direction opposite to tracking before the trial continued on (step ramp method, Rashbass, 1961). Grating stimuli gradually appeared and disappeared against a dark background according to a Gaussian temporal profile (SD: 300 ms). In each trial, observers were asked to indicate the presence/absence of a target grating as rapidly and accurately as possible by pressing a button. The duration between stimulus onset and the observer's judgment was taken as a measure of reaction time.

The accuracy of fixation tracking was verified for every trial by analyzing eye-tracking data. The analysis was done for the period from 0.5 to 2.5 s after stimulus onset until stimulus offset (3 s). The trajectory of the fixation point was first estimated by separately fitting a linear function to each eye's positional data. A root mean square error (RMSE) in X-Y position was calculated between the gaze and the estimated fixation point. This error was taken as a measure of the accuracy of fixation and tracking. We discarded trials in which the RMSE exceeded the 99% confidence interval of the average RMSE for a randomly chosen block of trials in the SFW condition for each observer. SD of the average RMSEs for the SFW condition was <1.2° across observers excepting one outlier observer and the discarding criteria thus only barely varied depending on individuals. As a result, 74% of trials were used for SFW, 76% were used for MF, 68% were used for MW, and 74% were used for MFW condition. A total of 73% (7270/9960) over all trials were used in the subsequent analysis.

### Reaction time analysis

After discarding all incorrect trials, 88% of target-present trials and 97% of target-absent remained and were used in the following analysis. An exponentially modified Gaussian (EMG) was fit to the cumulative distribution of reaction time by the least square method for each condition and observer. EMG is widely known to show a good fitness for analysis of reaction time (Hohle, 1965; Burbeck and Luce, 1982). EMG is expressed as follows:

(1) $$\begin{array}{l} {\text{Prop}}_{\text{CD}}=\frac{1+\text{erf}(\frac{\lambda (\text{RT }-\;\mu )}{\sqrt{2^{*}{\left(\lambda^{*}\sigma \right)}^{2}}})}{2}\\ \;-\exp\left[\begin{array}{l} -\lambda^{*}(\text{RT}-\mu )+\frac{{\left(\lambda^{*}\sigma \right)}^{2}}{2}\\ \;+\log(\frac{1\;+\text{ erf}(\frac{(\lambda (\text{RT}-\;\mu )\;-\;{\left(\lambda^{*}\sigma \right)}^{2}}{\sqrt{2^{*}{\left(\lambda^{*}\sigma \right)}^{2}}}}{2}) \end{array}\right] \end{array}$$

(2) $$erf(x)=\frac{2}{\sqrt{\pi }}\int_{0}^{x}e^{-t^{2}}dt$$

where μ, σ, λ are free parameters that indicate mean, SD, and exponential rate of EMG respectively. RT represents reaction time and Prop_CD_ represents proportion of cumulative distribution. From the function fit to EMG, we selected the time at which the proportion of cumulative distribution reached 0.5 for our representative value of reaction time.

## Results

**Figure 2:**
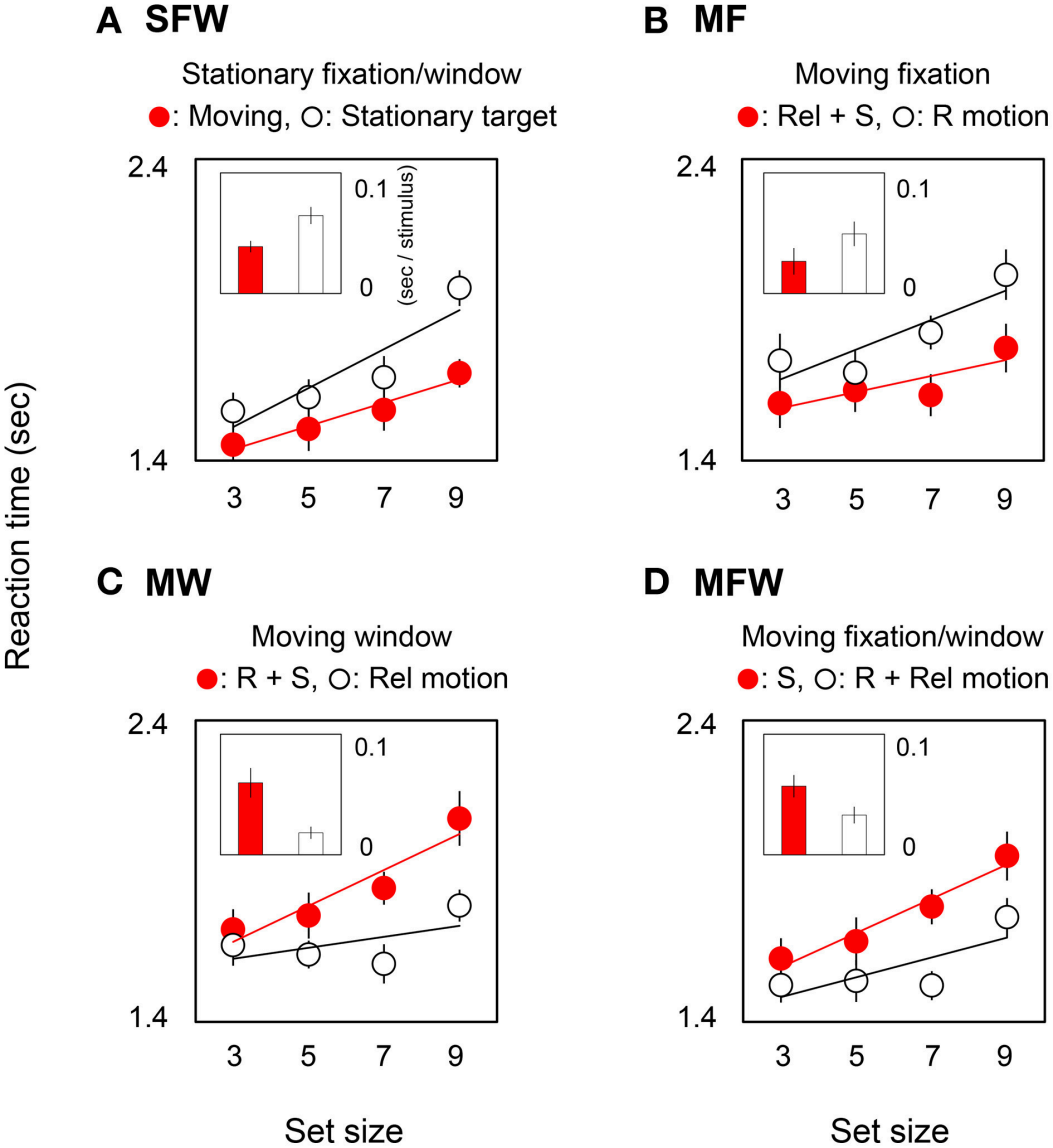
**Reaction times and slope values in target-present searches**. Each graph corresponds to the results for the four (**A**: SFW, **B**: MF, **C**: MW, **D**: MFW) conditions averaged across seven observers. Representative response times in the target presence/absence task (and a linear interpolation) are plotted as a function of set-size of grating stimuli. Each bar shows a slope value indicating search efficiency (sec/stimulus). Red and white plots show results for MOVING and STATIONARY targets respectively and include different combinations of motion signals across retinal (R), relative (Rel), and spatiotopic (S) coordinate systems. Error bars represent ±1 SE.

Figure 2 shows reaction times averaged across observers in target-present trials. In each graph, circles show reaction time as a function of set-size, and bars show the slopes of search functions as estimated by linear regression. Small and large slope values indicate efficient and inefficient searches respectively. Red circles and bars show results for detecting a MOVING target among STATIONARY distracters while white circles and bars show results for detecting a STATIONARY target among MOVING distracters. The four plots show result for the different combinations of moving/stationary parameters of the fixation point and spatial window or, equivalently, for different combinations of motion signals from retinal, relative, and spatiotopic coordinate systems.

Figure 2A shows results for the SFW condition. The reaction time for the MOVING target is noticeably shorter than for the STATIONARY target [ANOVA: *F*_(1, 6)_ = 11.25, *p* = 0.002], and this outcome is consistent with the classical moving/stationary target asymmetry found in visual search tasks (Royden et al., 2001).

Figure 2B shows results for the moving fixation (MF) condition wherein observers tracked a fixation point that moved along with the MOVING grating. As a consequence of visual tracking, the retinal motion present in the stationary fixation condition is eliminated, and the grating that remained stationary with respect to the window in the stationary fixation condition produces retinal motion. Thus, if the search asymmetry of moving/stationary targets is determined by retinal motion, the asymmetry should be reversed with respect to the SFW condition, and the reaction time to the STATIONARY target shown by the white circles should be shorter than the reaction time to the MOVING target shown by the red circles. Interestingly, however, the results indicate that this does not happen. Instead, the MOVING target was detected more rapidly than the STATIONARY target [ANOVA: *F*_(1, 6)_ = 9.05, *p* = 0.004]. These results clearly demonstrate that the search asymmetry of moving/stationary targets cannot be determined exclusively by retinal motion.

Figure 2C shows results for the moving window (MW) condition wherein the window moved along with the MOVING grating while observers fixated on a stationary point. In this condition, by virtue of moving windows, the relative motion of the originally moving (MOVING) grating is eliminated, and relative motion is generated for the STATIONARY grating. Importantly, however, the retinal and spatiotopic motions in this condition are identical to those in the SFW condition. Therefore, if retinal motion determines the search asymmetry, results should be the same as (or at least similar to) those obtained in the SFW condition. However, the STATIONARY target was detected more rapidly than the MOVING target [ANOVA: *F*_(1, 6)_ = 14.85, *p* = 0.0004]. This, again, shows that search asymmetry does not depend on retinal motion alone.

Figure 2D shows results for the MFW condition wherein the fixation point and the window moved along with the MOVING grating and observers tracked fixation. In this condition, retinal and relative motions are eliminated by visual tracking and the only motion signal generated by the originally moving (MOVING) grating is spatiotopic in nature. By comparison, as a result of fixation tracking, the originally STATIONARY grating generates retinal and relative motions signals but eliminates spatiotopic motion. Here, the STATIONARY target was detected faster than the MOVING target [ANOVA: *F*_(1, 6)_ = 13.14, *p* = 0.0007]. Across all conditions, reaction times tended to be shorter for stimuli that included relative motion signals.

**Figure 3:**
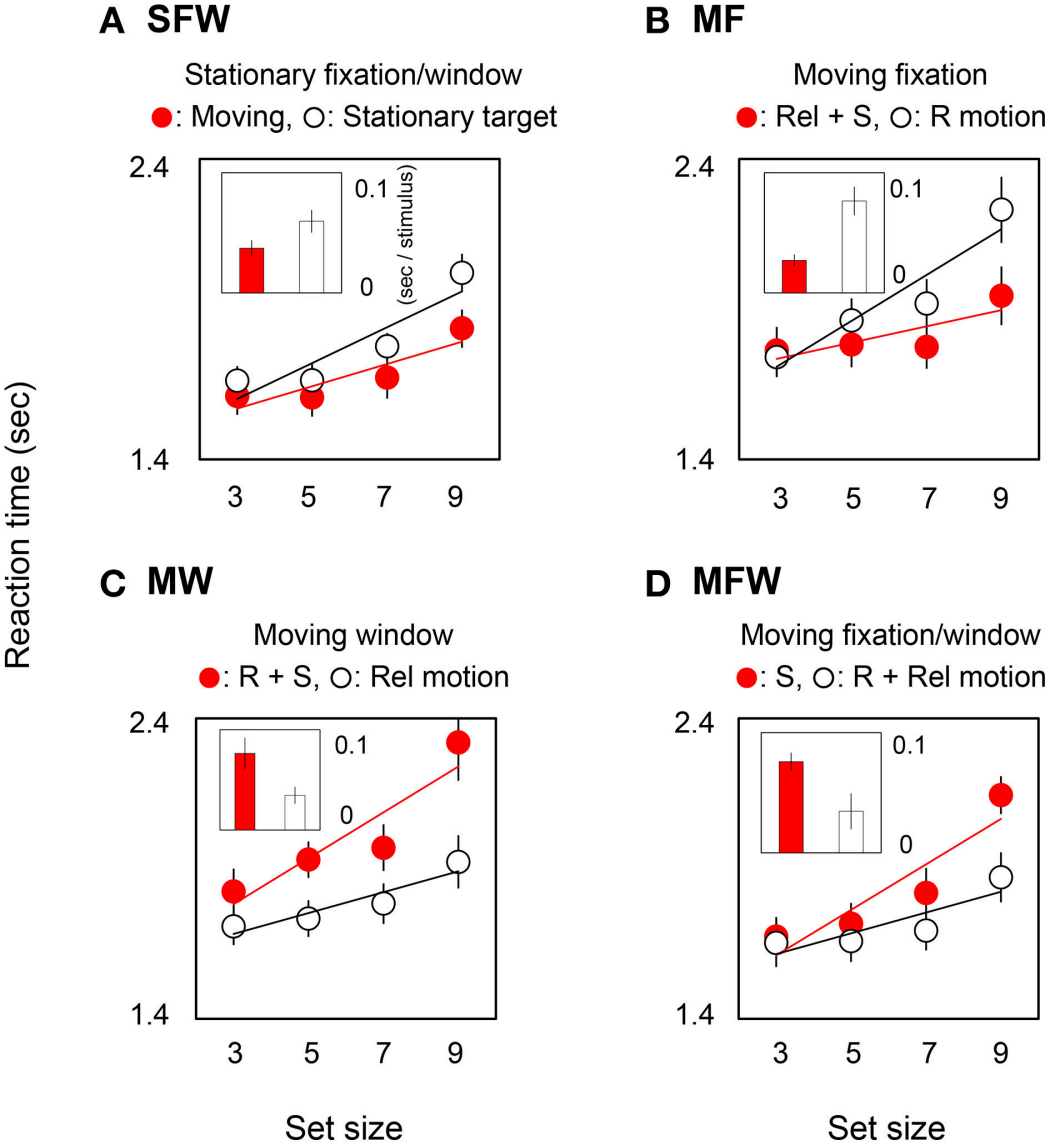
**Reaction times and slopes in target-absent searches for each (A: SFW, B: MF, C: MW, D: MFW) condition**. See explanation in Figure 2.

In order to quantify the effect of motion on search efficiency, we calculated the search slope for each of the MOVING and STATIONARY targets. For the SFW condition (Figure 2A), the slope value of the MOVING target shown by the red bar was lower than that of the STATIONARY target shown by the white bar (*t*-test: *p* = 0.01). The results indicate, as has been widely acknowledged (Verghese and Pelli, 1992; Royden et al., 2001), that a moving stimulus is detected more efficiently than a stationary stimulus. As for the MF condition (Figure 2B), slope values were not significantly different between the MOVING target that remained stationary on the retina by tracking and the STATIONARY target with retinal motion (*t*-test: *p* = 0.27). The results could be interpreted that search efficiency is not enhanced by retinal motion alone. For the MW condition (Figure 2C), slope values for MOVING targets are significantly higher than for STATIONARY targets (*t*-test: *p* = 0.02). While STATIONARY targets only produced relative motion with respect to the window that moved along with the MOVING target, MOVING targets had both retinal and spatiotopic motions. Results show that the asymmetry of search efficiency is determined not by retinal or spatiotopic motion but rather in large part by relative motion. In the MFW condition (Figure 2D), retinal and relative motions are generated for the originally stationary (STATIONARY) target. In this condition, slope values for STATIONARY targets are lower than for spatiotopically moving (MOVING) targets (*t*-test: *p* = 0.04). These data strongly suggest that the higher search efficiency for moving targets, when compared to search efficiency for stationary targets, is driven by relative motion signals. Figure 3 illustrates the reaction time averaged across observers in the target-absent trials and reveals a similar moving-vs.-stationary target search asymmetry for reaction times and slope values.

### The relative contribution of motion signals from different coordinate systems

In this section, we seek to quantify the relative contribution of motion signals from three important coordinate systems (retinal, relative, and spatiotopic) by using a weighted linear summation model analogous to models used for investigating the effect of different cues for depth perception (Clark and Yuille, 1990; Johnston et al., 1994; Landy et al., 1995). It is generally known that search efficiency is diminished if target and distracter are similar but enhanced if distracters are similar to each other (Duncan and Humphreys, 1989; Nothdurft, 1993; Rosenholtz, 1999).

Our model is based on the assumption that motions from different coordinate systems are represented separately by their weighted velocities and that search efficiency is determined by weighted-velocity summation. Accordingly, slope value would be inversely proportional to the distance between target and distracter in three-dimensional velocity space of retinal, relative, and spatiotopic coordinates but proportional to the variance of these velocities in distracter:

(3) $$\begin{array}{l} Slope=\frac{Variance(W_{R}{\;}^{*}R_{D},W_{Rel}{\;}^{*}Rel_{D},W_{S}{\;}^{*}S_{D})}{{\left\{\begin{array}{l} {\left(W_{R}{\;}^{*}R_{T}-W_{R}{\;}^{*}R_{D}\right)}^{2}+(W_{Rel}{\;}^{*}Rel_{T}-W_{Rel}\\ \;{\;}^{*}Rel_{D}{)}^{2}+{\left(W_{S}{\;}^{*}S_{T}-W_{S}{\;}^{*}S_{D}\right)}^{2} \end{array}\right\}}^{1/2}}\\ \;+Base\_lope \end{array}$$

where *R*_*T*_, Re*l*_*T*_, *S*_*T*_ are binary variables that indicate the presence (1) or absence (0) of motion in a given coordinate system (retinal, relative, or spatiotopic) for the target, and similarly, *R*_*D*_, Re*l*_*D*_, *S*_*D*_ represent the presence/absence of motion for distracters. *W*_*R*_, *W*_*Rel*_, and *W*_*S*_ are free parameters that specify the weight of each motion coordinate system, and we estimated these weights by optimizing the model's fit between model prediction and actual data for each observer.

**Figure 4:**
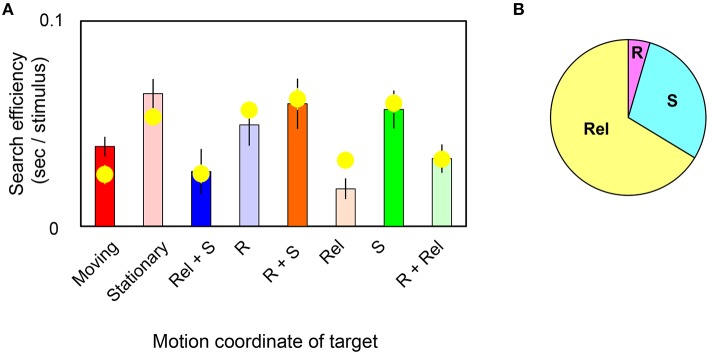
**(A)** Each bar shows the slope value of the MOVING/STATIONARY target for four different motion conditions in target-present trials. Yellow circles represent the average of the slope value as estimated by the weighted-sum model. Error bars represent ±1 SE across observers. **(B)** Pieplot shows the relative weights of retinal (R), relative (Rel), and spatiotopic (S) motions as estimated by the weighted-sum model.

In Figure 4A, bars replot the observed slope values and yellow circles represent slope values estimated by the model in target-present trials. The correlation coefficient was found to be 85% between them. Estimated weights are (*W*_R_, *W*_Rel_, *W*_S_) = (0.01, 0.12, 0.05; s.e.m. = 0.01, 0.02, 0.02 across observers). The pieplot in Figure 4B shows the relative weights for motions defined by retinal (R), relative (Re*l*), and spatiotopic (S) coordinates. These weights mean that relative motion significantly enhances the search efficiency (*t*-test, *p* = 0.001) and that spatiotopic motion makes a more modest contribution (*t*-test, *p* = 0.07). However, retinal motion contributes little overall (*t*-test, *p* = 0.20). These results quantitatively indicate that visual search for moving/stationary targets does not involve retinal motion but instead depends largely on relative-motion signals, and to a lesser degree on spatiotopic motion signals, in target stimuli.

## Discussion

The present study examined how types of motion signals defined in different coordinate systems—retinal, relative, and spatiotopic—generate the search asymmetry effect found for moving/stationary targets. Data indicate that the asymmetry is not explained by retinal motion signals but is instead driven largely by relative motion signals which, in turn, determine the direction of the search asymmetry. This finding holds true for measured reaction time and search efficiency both for target-present and target-absent trials. The quantitative analyses we have performed also reveal that, over and above the unique contribution of relative motion signals, spatiotopic motion signals contribute substantially to the efficient search for moving targets but that retinal motion hardly factors in at all. These results show that, perhaps unintuitively, the saliency of visual motion in visual search predominantly depends on extra-retinal information.

It is widely known that moving stimuli pop out among stationary stimuli and, in this case, the slope of the search function approaches zero (Treisman and Gelade, 1980; Treisman, 1986). In the present study, however, slope values were larger than zero for all the conditions tested. These slope values indicate that the detection task depended at least partly on a serial search process commonly believed to require focal attention (Dick et al., 1987). We found the shallowest slope value to be in the MW condition wherein targets only produced relative motion, and serial search (i.e., the steepest slope) was most likely involved in the SFW condition in which moving targets contain all types of motions (Figure 4A). Observers may have been attending preferentially to the relative motion component, but the coexistence of retinal or spatiotopic motion with relative motion might have interfered with an efficient search process. As a whole, our study suggests that attentional processes mediate the detection of targets defined by non-retinal motions. This is also consistent with previous studies indicating that, in a cued search, observer's attention could be guided by object-centered positions rather than a classical retinotopic map (Boi et al., 2009, 2011; Theeuwes et al., 2013).

In a study on smooth pursuit eye movements, Morvan and Wexler (2005) reported higher detection rates for a target moving more rapidly than distracters than for a target moving more slowly than distracters but that dependency of this effect on retinal or physical speed had changed critically by stimulus duration: the effect depended on the retinal speed if stimulus presentation was shorter than 130 ms, but the asymmetry reversed itself in accordance with the physical speed if stimuli were presented for longer than 150 ms. One might expect that neural computations involved in calculating spatiotopic motion take place higher up the visual-processing hierarchy than those responsible for retinal and relative motion signals (Wurtz, 2008) and that, in such cases, one would expect the contribution of spatiotopic motion to the search asymmetry to take longer presentation times to manifest itself. To examine this possibility, we calculated the correlation between reaction time and contribution of motions in different coordinates across observers for the present results. The correlation coefficient was found to be -0.47 in retinal, 0.09 in relative, 0.42 in spatiotopic coordinates. These correlation values imply that the contribution of retinal motion is smaller for observers who respond more slowly and that the contribution of spatiotopic motion is larger for these same observers. This finding is in keeping with the notion that the spatiotopic coordinate system requires higher-level (and therefore slower) processing to integrate retinal inputs and sensorimotor signals from the eyes and/or body. It is otherwise difficult to directly compare the present results with those of Morvan and Wexler (2005) since stimuli (gratings and dots respectively) and the type of search task (reaction time and detection rate respectively) differed significantly between the two studies. In addition, the effects of spatiotopic motion described in Morvan and Wexler (2005) could be accounted for by relative motion with respect to the visible frame of the display in their study. Recall from our methods (see Section Procedure) that we ensured the frame of our display remained nearly invisible in order to keep the contribution of extraneous relative motion cues (e.g., autokinetic sensation, Adams, 1912; induced motion, Duncker, 1929) to a strict minimum.

The present findings could also be interpreted as indicating that the saliency of moving stimuli is produced by means of a mechanism that processes non-retinal motions. Several studies discussed the relation between the spatiotemporal property of this saliency and non-retinal coordinates. In binocular rivalry, for instance, van Boxtel et al. (2008) demonstrated that the visibility of a pattern was affected if it was presented at the same spatiotopic position as its prior adaptation across the observer's eye movement. Recently, we also explored the motion dominance in binocular rivalry using a similar manipulation to the present study (Nakayama et al., 2016). That particular study showed that the contribution of relative motion increases but that of retinal motion decreases as the luminance contrast is increased, and that spatiotopic motion makes a significant but constant contribution. It is plausible that, in the present study, the neural representation of the stimulus window signaled by luminance modulation was distinct and facilitated relative-motion processing in manner similar to the contrast effects observed in Nakayama et al. (2016) and other psychophysical studies (Zhang et al., 1993; Tadin et al., 2003). Although it is difficult to identify and directly compare factors contributing to distinct visual processes, results from our visual search and binocular rivalry studies point to the idea that attentional selection and/or perceptual consciousness of visual stimuli is involved in the higher-order processing in non-retinal coordinates.

In the present study, observers could potentially have detected the presence/absence of relative motion between target and distracter. This relative motion cannot be separated from the search task since the target itself was defined by difference of moving/stationary parameter. Note, however, that this relative motion component was included throughout all experimental conditions in exactly the same way and therefore cannot be invoked as an explanation for the search asymmetry reported herein. Further, a possibility that the present results depended on temporal frequency is strictly limited as a search for flickering targets defined only by difference of temporal frequency has been known to be symmetric (Cass et al., 2011). It is therefore more likely that the enhancements measured in reaction time and search efficiency arose from experimental manipulations that directly controlled the respective strength of motion signals from retina, relative, and spatiotopic coordinate systems.

## Author contributions

RN and IM conceived the study. RN designed and performed experiments, analyzed data, and drafted the manuscript. IM and TS supervised the project and helped drafting the manuscript.

### Conflict of interest statement

The authors declare that the research was conducted in the absence of any commercial or financial relationships that could be construed as a potential conflict of interest.

## References

[B1] Abrams R. A. Christ S. E. (2006). Motion onset captures attention: a rejoinder to Franconeri and Simons (2005). Percept. Psychophys. 68, 114–117. 10.3758/BF03193661 16617835

[B2] Adams H. (1912). Autokinetic sensations. Psychol. Monogr. 14, 1–45. 10.1037/h0093066

[B3] Allman J. Miezin F. McGuinness E. (1985). Stimulus specific responses from beyond the classical receptive field: neurophysiological mechanisms for local-global comparisons in visual neurons. Annu. Rev. Neurosci. 8, 407–430. 10.1146/annurev.ne.08.030185.002203 3885829

[B4] Aubert H. (1887). Die bewegungsempfindung. Pfliigers Arch. Ges. Physiol. 40, 459–480. 10.1007/BF0161271026527229

[B5] Boi M. Öğmen H. Krummenacher J. (2009). A (fascinating) litmus test for human retino- vs non-retinotopic processing. J. Vis. 9, 1–11. 10.1167/9.13.5 20055538 PMC2904816

[B6] Boi M. Vergeer M. Ogmen H. Herzog M. H. (2011). Nonretinotopic exogenous attention. Curr. Biol. 21, 1732–1737. 10.1016/j.cub.2011.08.059 22000104 PMC3408210

[B7] Born R. T. Tootell R. B. H. (1992). Segregation of global and local motion processing in primate middle temporal visual area. Nature 357, 497–499. 10.1038/357497a0 1608448

[B8] Brainard D. H. (1997). The psychophysics toolbox. Spat. Vis. 10, 433–436. 10.1163/156856897X00357 9176952

[B9] Burbeck S. L. Luce R. D. (1982). Evidence from auditory simple reaction times for both change and level detectors. Percept. Psychophys. 32, 117–133. 10.3758/BF03204271 7145582

[B10] Cass J. Van der Burg E. Alais D. (2011). Finding flicker: critical differences in temporal frequency capture attention. Front. Psychol. 2:320. 10.3389/fpsyg.2011.00320 22110460 PMC3216028

[B11] Chukoskie L. Movshon J. A. (2009). Modulation of visual signals in macaque MT and MST neurons during pursuit eye movement. J. Neurophysiol. 102, 3225–3233. 10.1152/jn.90692.2008 19776359 PMC2804434

[B12] Clark J. J. Yuille A. L. (1990). Fusing binocular and monocular depth cues, in Data Fusion for Sensory Information Processing Systems. Engineering and Computer Science, Kluwer International Series, eds Clark J. J. Yuille A. L. (Boston, MA: Kluwer Academic Publishers), 137–146.

[B13] Dick M. Ullman S. Sagi D. (1987). Parallel and serial processes in motion detection. Science 237, 400–402. 10.1126/science.3603025 3603025

[B14] Duncan J. Humphreys G. W. (1989). Visual search and stimulus similarity. Psychol. Rev. 96, 433–458. 10.1037/0033-295X.96.3.433 2756067

[B15] Duncker K. (1929). Über induzierte Bewegung. Psychol. Forsch. 12, 180–259. 10.1007/BF02409210

[B16] Erickson R. G. Thier P. (1991). A neuronal correlate of spatial stability during periods of self-induced visual motion. Exp. Brain Res. 86, 608–616. 10.1007/BF00230534 1761094

[B17] Filehne W. (1922). Uber das optische Wahrnehmen von Bewegungen. Z. Sinnephysiol. 53, 134–145.

[B18] Fleischl E. V. (1882). Physiologiseh-optisehe. Notizen. 2. Mitt. S. B. Akad. Wiss. Wien 86(Abt. 3), 8–25.

[B19] Galletti C. Battaglini P. P. Fattori P. (1990). “Real-motion”cells in area V3A of macaque visual cortex. Exp. Brain Res. 82, 67–76. 10.1007/BF00230838 2257915

[B20] Hillstrom A. P. Yantis S. (1994). Visual-Motion and attentional capture. Percept. Psychophys. 55, 399–411. 10.3758/BF03205298 8036120

[B21] Hohle R. H. (1965). Functions of foreperiod duration, 69, 382–386. 10.1037/h0021740 14286308

[B22] Johnston E. B. Cumming B. G. Landy M. S. (1994). Integration of stereopsis and motion shape cues. Vision Res. 34, 2259–2275. 10.1016/0042-6989(94)90106-6 7941420

[B23] Landy M. S. Maloney L. T. Johnston E. B. Young M. (1995). Measurement and modeling of depth cue combination: in defense of weak fusion. Vision Res. 35, 389–412. 10.1016/0042-6989(94)00176-M 7892735

[B24] Morvan C. Wexler M. (2005). Reference frames in early motion detection. J. Vis. 5, 131–138. 10.1167/5.2.4 15831073

[B25] Nakayama R. Motoyoshi I. Sato T. (2016). Motion dominance in binocular rivalry depends on extra-retinal motions. J. Vis. 16, 1–10. 10.1167/16.5.2 26943347

[B26] Nothdurft H. C. (1993). The role of features in preattentive vision: comparison of orientation, motion and color cues. Vision Res. 33, 1937–1958. 10.1016/0042-6989(93)90020-W 8249312

[B27] Pelli D. G. (1997). The videotoolbox software for visual psychophysics: transforming numbers into movies. Spat. Vis. 10, 437–442. 10.1163/156856897X00366 9176953

[B28] Rashbass C. (1961). The relationship between saccadic and smooth tracking eye movements. J. Physiol. 159, 326–338. 10.1113/jphysiol.1961.sp006811 14490422 PMC1359508

[B29] Rosenholtz R. (1999). A simple saliency model predicts a number of motion popout phenomena. Vision Res. 39, 3157–3163. 10.1016/S0042-6989(99)00077-2 10615487

[B30] Royden C. S. Wolfe J. M. Klempen N. (2001). Visual search asymmetries in motion and optic flow fields. Percept. Psychophys. 63, 436–444. 10.3758/BF03194410 11414131

[B31] Sakata H. Shibutani H. Kawano K. Harrington T. L. (1985). Neural mechanisms of space vision in the parietal association cortex of the monkey. Vision Res. 25, 453–463. 10.1016/0042-6989(85)90070-7 4024464

[B32] Schütz A. C. Braun D. I. Kerzel D. Gegenfurtner K. R. (2008). Improved visual sensitivity during smooth pursuit eye movements. Nat. Neurosci. 11, 1211–1216. 10.1038/nn.2194 18806785

[B33] Schütz A. C. Delipetkos E. Braun D. I. Kerzel D. Gegenfurtner K. R. (2007). Temporal contrast sensitivity during smooth pursuit eye movements. J. Vis. 7, 1–15. 10.1167/7.13.3 17997631

[B34] Tadin D. Lappin J. S. Gilroy L. A. Blake R. (2003). Perceptual consequences of centre-surround antagonism in visual motion processing. Nature 424, 312–315. 10.1038/nature01800 12867982

[B35] Terao M. Murakami I. (2011). Compensation for equiluminant color motion during smooth pursuit eye movement. J. Vis. 11, 1–12. 10.1167/11.6.12 21602556

[B36] Theeuwes J. (1994). Stimulus-driven capture and attentional set: selective search for color and visual abrupt onsets. J. Exp. Psychol. Hum. Percept. Perform. 20, 799–806. 10.1037/0096-1523.20.4.799 8083635

[B37] Theeuwes J. (1995). Temporal and spatial characteristics of preattentive and attentive processing. Vis. Cogn. 2, 221–233. 10.1080/13506289508401732

[B38] Theeuwes J. Mathôt S. Grainger J. (2013). Exogenous object-centered attention. Atten. Percept. Psychophys. 75, 812–818. 10.3758/s13414-013-0459-4 23636948

[B39] Treisman A. (1986). Features and objects in visual processing. Sci. Am. 255, 114–125. 10.1038/scientificamerican1186-114B

[B40] Treisman A. Gelade G. (1980). A feature-integration theory of attention. Cogn. Psychol. 12, 97–136. 10.1016/0010-0285(80)90005-5 7351125

[B41] van Boxtel J. J. Alais D. van Ee R. (2008). Retinotopic and non-retinotopic stimulus encoding in binocular rivalry and the involvement of feedback. J. Vis. 8, 1–10. 10.1167/8.5.17 18842088

[B42] Verghese P. Pelli D. (1992). The information capacity of visual attention. Vision Res. 32, 983–995. 10.1016/0042-6989(92)90040-P 1604866

[B43] Wolfe J. M. (2001). Asymmetries in visual search: an introduction. Percept. Psychophys. 63, 381–389. 10.3758/BF03194406 11414127

[B44] Wurtz R. H. (2008). Neuronal mechanisms of visual stability. Vision Res. 48, 2070–2089. 10.1016/j.visres.2008.03.021 18513781 PMC2556215

[B45] Zhang J. Yeh S. L. De Valois K. K. (1993). Motion contrast and motion integration. Vision Res. 33, 2721–2732. 10.1016/0042-6989(93)90231-K 8296468

